# Hypercalcemia in a patient with disseminated paracoccidioidomycosis: a case report

**DOI:** 10.1186/1752-1947-2-262

**Published:** 2008-08-08

**Authors:** Rafael Moura Almeida, Loureno Cezana, Daniela Miti Lemos Tsukumo, Marco Antônio de Carvalho-Filho, Mário José Abdalla Saad

**Affiliations:** 1 Department of Internal Medicine, State University of Campinas, 13081-970 Campinas, SP, Brazil

## Abstract

**Introduction:**

Hypercalcemia is well described in various granulomatous disorders, such as sarcoidosis, tuberculosis, berylliosis, leprosy and fungal infections. However, the association of *Paracoccidioides brasiliensis *and hypercalcemia is rare: to the best of our knowledge, only two cases have previously been reported, and neither had a clear documentation of the etiology of the hypercalcemia.

**Case presentation:**

We report the case of a 22-year-old man in whom disseminated infection with paracoccidioidomycosis was associated with hypercalcemia. The patient had a high normal serum level of 1,25-dihydroxyvitamin D and a suppressed parathyroid hormone value, an indication that the hypercalcemia was not mediated by parathyroid hormone and might be associated with 1,25-dihydroxyvitamin D.

**Conclusion:**

The episode resolved readily with administration of corticosteroids, an outcome suggesting that this is an effective treatment of hypercalcemia of this origin. On follow-up, while receiving antifungal therapy for *P. brasiliensis *the patient's calcium values remained normal.

## Introduction

Hypercalcemia is well described in various granulomatous disorders, such as sarcoidosis, tuberculosis, berylliosis, leprosy and fungal infections. Among the fungal diseases, disseminated candidiasis, histoplasmosis, cryptococcosis and coccidioidomycosis have rarely been implicated as causes of hypercalcemia [1-5]. In this report we describe a patient in whom hypercalcemia was associated with disseminated infection with *Paracoccidioides brasiliensis *and a high normal serum level of 1,25-dihydroxyvitamin D.

## Case presentation

The patient, a 22-year-old male agriculturalist was admitted to our hospital in November 2006 with asthenia, weight loss, fever, diffuse enlargement of cervical lymph nodes and enlarged liver and spleen. The patient's temperature was 38.5°C and his blood pressure was 120/80 mmHg with a pulse of 96 beats per minute.

Initial laboratory studies included an erythrocyte sedimentation rate of 89 mm/hour, serum hemoglobin of 11.4 g/dl, white blood cell count of 13,690/mm^3 ^(27% eosinophils), serum albumin of 2.4 g/dl, normal serum urea, creatinine, sodium and potassium. On admission, the patient had a total serum calcium level of 10.4 mg/dl (normal range 8.4 to 9.7 mg/dl; ~12 mg/dl when corrected for albumin), ionized calcium of 1.46 mmol/l (normal range 1.15 to 1.29 mmol/l) and phosphorus of 4.9 mg/dl (normal range 2.7 to 4.5 mg/dl). An aspiration of the cervical lymph node demonstrated *P. brasiliensis*.

Results of other laboratory tests performed to help assess the cause of the hypercalcemia were as follows: thyroid stimulating hormone 4.51 IU/ml (normal range 0.41 to 4.5 IU/ml); parathyroid hormone (PTH) 3.4 pg/ml (normal range 15 to 65 pg/ml); 1,25-dihydroxyvitamin D 49.4 pg/ml (normal range 15.9 to 55.6 pg/ml); and 24-hour urinary calcium 856.55 mg (normal range 100 to 300 mg/24 hours). Chest X-ray and bone scans were normal. A computed tomography scan of the chest and abdomen revealed no underlying malignancy. Other causes of hypercalcemia such as vitamin A and D intoxication, sarcoidosis, multiple myeloma, milk-alkali syndrome, adrenal insufficiency and immobilization were excluded on the basis of laboratory and clinical data.

Despite the administration of hydration with normal saline and furosemide therapy, the patient's calcium level increased to 1.59 mmol/l. Specific treatment for paracoccidioidomycosis was initiated with trimethoprim-sulfamethoxazole. At that time, prednisone (20 mg/day) was added to the regimen. On the 13th day of hospitalization, the ionized calcium level had decreased to 1.34 mmol/l and on the day of discharge from hospital this level was 1.26 mmol/l. The patient received follow-up examinations until May 2007 and during this time has been asymptomatic, with normal levels of serum calcium and 1,25-dihydroxyvitamin D (22.4 pg/ml).

## Discussion

We have reported the case of a patient presenting with hypercalcemia complicating disseminated infection with paracoccidioidomycosis. Hypercalcemia is well described in various granulomatous disorders; however, the association of *P. brasiliensis *and hypercalcemia is rare. To the best of the authors' knowledge, only two cases have been reported previously, neither of which offered a clear documentation of the etiology of the hypercalcemia [6,7].

The endogenous overproduction of 1,25-dihydroxyvitamin D by activated macrophages seems to have a central causative role in some granuloma-forming diseases, particularly sarcoidosis [8], although it is not uniformly observed [5,8]. In our patient, the high normal levels of 1,25-dihydroxyvitamin D suggest that it may have had a role in the hypercalcemia.

Our patient demonstrated total and ionized hypercalcemia, associated with low serum PTH, elevated serum phosphorous and normal renal function. The elevation of 1,25-dihydroxyvitamin D (high normal limit) was unusual for the suppressed PTH and elevated phosphorous levels, suggesting an inappropriately elevated production or decreased clearance of 1,25-dihydroxyvitamin D [9]. The hypercalcemia was initially treated with hydration and furosemide, without improvement. Hypercalcemia normalized when prednisone 20 mg was initiated. Although antifungal therapy may have contributed to a partial improvement of the hypercalcemia, a more likely explanation is that the prompt response was due to treatment with prednisone. Thus, 1,25-dihydroxyvitamin D might have a role in the pathogenic mechanism of hypercalcemia in paracoccidioidomycosis.

## Conclusion

We have described a patient in whom hypercalcemia was associated with disseminated infection with *P. brasiliensis *and a high normal serum level of 1,25-dihydroxyvitamin D.

## Abbreviations

PTH: Parathyroid hormone.

## Competing interests

The authors declare that they have no competing interests.

## Authors' contributions

RMA, LC and DMLT contributed to the care of the patient, were involved in the preparation of the manuscript and undertook the medical literature search. DMLT, MACF and MJAS were responsible for patient's management. MJAS was involved in the conception of the article and revised it critically for important intellectual data before final approval. All authors read and approved the final manuscript.

## Consent

Written informed consent was obtained from the patient for publication of this case report and accompanying images. A copy of the written consent is available for review by the Editor-in-Chief of this journal.
